# Synthesis and characterization of PEDSCD and its application as a flame retardant in epoxy resins

**DOI:** 10.1039/d1ra06116j

**Published:** 2021-10-27

**Authors:** Yi Zhang

**Affiliations:** Department of Biology and Chemical Engineering, Shandong Vocational College of Science & Technology Weifang 261053 China emmazhangling@163.com

## Abstract

In this study, a flame retardant agent, called PEDSCD, is synthesized through a polycondensation reaction. PEDSCD is a chemically expanded phosphorus-containing flame retardant, which is introduced in epoxy resin (EP) to improve its flame retardancy. The molecular structure and thermal stability of PEDSCD are characterized by nuclear magnetic resonance, Fourier transform infrared spectroscopy, and thermogravimetric analysis, and EP/PEDSCD composites are investigated in detail. EP/PEDSCD exhibits good stability and flame retardancy. These properties are attributed to the triazine structure introduced into the flame retardant system. The triazine structure starts to decompose at a lower temperature and also reacts with the phosphorus element to form P=N–, which increases the viscosity of the melt. This inhibits the generation of smoke and reduces the peak of heat release. PEDSCD shows good thermal stability and low flammability. Further, the weight loss from 500 to 800 °C is only 16 wt%, and the residual mass at 800 °C is 32 wt%. With the addition of PEDSCD, the flame retardant quality of the EP/PEDSCD composites is gradually enhanced, and the carbon residue becomes denser, which isolates the heat transfer and inhibits the volatilization of flue gas. The limited oxygen index (LOI) value of 27% and a vertical burning V-0 rating are achieved when PEDSCD is used in combination with ammonium polyphosphate (APP). The cone calorimeter test shows that the peak heat release rate is reduced by 29% and low gas content is generated, which verifies that the combination of PEDSCD and other phosphorus-containing flame retardants exhibits significantly enhanced flame-retardant properties. PEDSCD exhibits a charring and barrier effect in the condensation phase. Overall, using basic characterization and flame retardancy testing, it is proved that PEDSCD exhibits good flame retardancy when added to EP.

## Abstract 2

In this study, a flame retardant agent, called PEDSCD, is synthesized through a polycondensation reaction.

## Graphical abstract

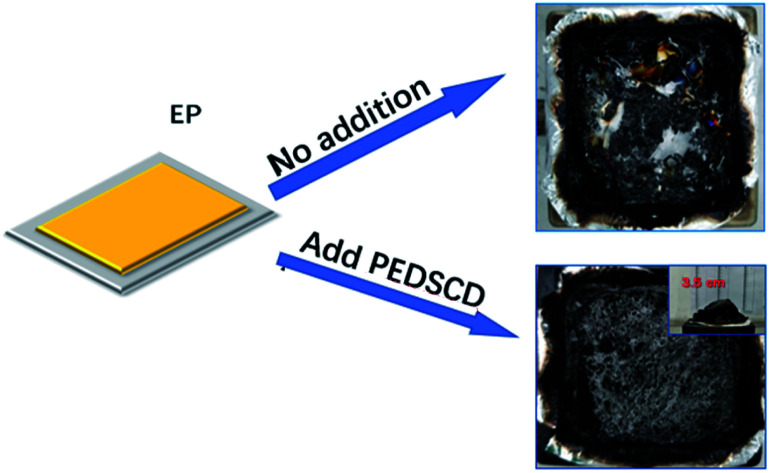

## 1. Introduction

Epoxy resin (EP) is widely used in the fields of electrical appliances, adhesives, anti-corrosive coatings, and composite materials owing to its excellent mechanical properties, low cost, low shrinkage, high specific strength, good dimensional stability, super adhesiveness and good chemical and thermal resistance. However, EP is a flammable material with a limited oxygen index (LOI) of only 19%. Thus, EP has poor flame retardancy and releases a large amount of heat and volatile substances during combustion, which threatens life and property, thereby limiting the practical application of EPs. Therefore, there is a growing need for the preparation of flame retardant EPs by the introduction of additives.

Among the various flame retardants, chemical intumescent flame retardants (IFRs) containing an acid source, a gas source, and a carbon source have received extensive attention due to their excellent flame retardancy. The IFRs are usually generated from phosphorus–nitrogen compounds. The phosphorus-containing compounds are used as acid sources, which can promote polymer loss of water and improve the quality of polymer residues after combustion, while nitrogen-containing compounds affect the combustion process of polymer materials. The non-flammable gases such as ammonia or nitrogen are released, which dilute the flammable gas and promote the viscous polymer material to form a foam expansion layer. Nitrogen-containing compounds used in IFRs, such as foamed carbon source–triazine structure, can improve the char formation effect and increase the release of non-combustible gases in the system during the flame retardancy process. These compounds play the role of gas source and supplementary carbon source.^1–3^ Ma *et al.*^4^ used phosphorus oxychloride, pentaerythritol, and 4,4-diaminodiphenylmethane (DDM) to synthesize a P–N–containing oligomeric IFR, named poly(diaminodiphenyl methane spirocyclic pentaerythritol bisphosphonate) (PDSPB). It was found that the PDSPB rapidly expanded into carbon when heated. The PO–Ph and aromatic/graphite layer formed at 400–450 °C greatly increased the stability of the carbon layer and provided good protection for the material. However, more heat and smoke were released. Song *et al.*^5^ used POCl_3_, PER, and DDM as raw materials to synthesize a single-component IFR, namely poly(4,4′-diamino-diphenyl methane-*O*-bicyclopentaerythritol phosphate–phosphate) (PDBPP). PDBPP had good thermal stability and char-forming ability, but due to insufficient gas source, it had a poor flame retardant effect when used alone. Zuo *et al.*^6^ used cyanuric chloride, morpholine, and triethyl phosphite to prepare a phosphorus and nitrogen-containing three-source IFR, called triazine oligomer poly (2-morpholinyl-4-pentaerythritol phosphate-1,3,5-triazine) (PMPT). It was used to improve the flame retardancy of polypropylene (PP). Compared with that of PP, the LOI of PP/30 wt% PMPT flame retardant composite increased by nearly 59.7% and it achieved UL-94 V-0 rating. However, due to the excessive PMPT gas source, the carbon layer after combustion was loose, and the cells were too large. The synthesis of two kinds of triazine flame retardants results in a poor flame retardant effect. Accordingly, the development of a new type of chemical IFR that not only has a good flame retardant effect but also can effectively reduce the release of smoke and heat is urgently needed. The traditional chemical intumescent flame retardant system, in the process of use, there are such as between the components and the polymer. The phase interface compatibility between the phases is not good, and when the amount is too large, it is easy to cause problems such as the decrease of the mechanical properties of the material.

In this work, triethylamine is used as an acid binding agent to react with phosphate ester (PEPA) and cyanuric chloride in anhydrous acetonitrile to prepare cage-like phosphate–triazine compound PECD, which is then condensed with amino curing agent DDS in acetonitrile to synthesize a cage-like PEPA is an oligomeric flame retardant with a triazine structure in the main chain: poly4,4-diamino-*p*-phenylenedisulfone-1,3,5-*s*-triazine-O*-*l-oxo-4-hydroxymethyl-2,6,7-trioxa-1-phosphabicyclo [2.2.2] octane (PEDSCD). Three flame retardants are tested using Fourier transform infrared (FTIR) spectroscopy and nuclear magnetic resonance (NMR) to characterize the structure of the synthesized oligomers, and thermogravimetric analysis (TGA) is used to examine their thermal stability. The synthetic method of the flame retardant synthesized in this article is simple, and the post-processing of the synthesized oligomer is easy. When used for EP flame retardant, it can achieve a better reduction at a lower addition amount and a lower phosphorus content. The total amount of heat release and the flame retardant effect of reducing the peak heat release furthermore, PEDSCD and APP are used in combination, and its flame retardant performance is compared with that of pure EP and EP/PEDSCD composites.

## 2. Experimental

### 2.1 Materials

Pentaerythritol (PER) was purchased from Beijing Chemical Plant (Beijing, China). Phosphorus oxychloride was purchased from Tianjin Guangfu Fine Chemical Research Institute (Tianjin, China). 4,4-Diaminodiphenylsulfone (DDS), 4,4-diaminodiphenylmethane (DDM), and dimethylsulfoxide (DMSO) were purchased from Sinopharm Chemical Reagent Beijing Co., Ltd (Beijing, China). Triethylamine and acetonitrile were purchased from Beijing Chemical Plant (Beijing, China). Melamine was purchased from Sann Chemical Technology (Shanghai) Co., Ltd (Shanghai, China). PEPA was made in the laboratory. Epoxy resin (DGEBA, commercial name: E-44) was supplied by Sinopec Baling Petrochemical Branch (Yueyang, China). All materials were of reagent grade and were used as received.

### 2.2 Synthesis of intermediate PEPA

**Fig. 1:**
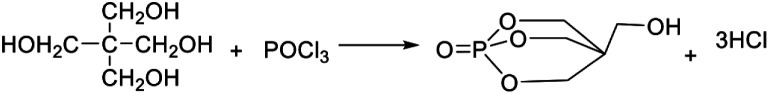
Synthetic route of PEPA (dioxane).

The synthetic route of PEPA is shown in Fig. 1. Add 68 g (0.5 mol) of pentaerythritol into a mechanical stirrer, add 200 ml of dioxane to the 500 ml three-necked flask of the condenser, stir to dissolve, and use the dropping funnel to add dropwise. 20 ml of phosphorus oxychloride, keep stirring, heat up to 80 °C at a rate of 5 °C min^−1^, and then use a drop to leak within four hours drop 26.73 ml (total 0.51 mol) of phosphorus oxychloride into the bucket. The acid gas emitted during the reaction is absorbed by lye. Opposite the system should first change from clarification to turbidity, and then from turbidity to clarification. The temperature is raised to 90 °C, and the reaction is 1 h, there is a white solid after 5 h of continuous refluxing, no acid gas will be precipitated. Cool the system to room temperature and filter with suction to obtain a crude solid product things. Wash the crude product three times with anhydrous ethanol and acetone successively, filter with suction to obtain a white powder, and place the white powder in the dry in a vacuum oven at 80 °C for 8 hours to obtain a white PEPA powder with a yield of 75.7%.^7–9^

### 2.3 Synthesis of intermediate PECD

**Fig. 2:**
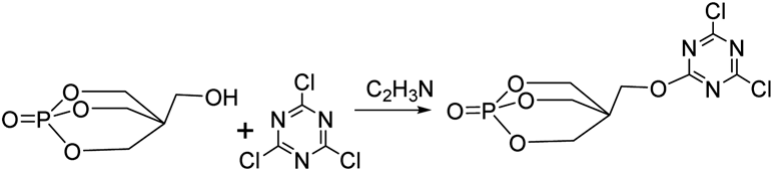
Synthetic route of PECD.

The synthetic route of the intermediate PECD is shown in Fig. 2. Under nitrogen protection, 18.4 g (0.1 mol) cyanuric chloride, 29.7 g (0.1 mol) PEPA, and 300 ml acetonitrile were added in a 500 ml three-necked flask equipped with a mechanical stirrer and reflux condenser. The solution was stirred until the PEPA was completely dissolved. Subsequently, the temperature was raised to 110 °C, and the heating was stopped after 24 h of reaction. After evaporating most of the acetonitrile by rotating the transparent liquid, the white crystals obtained were rinsed with acetone for three times. The crystals were then placed in a vacuum oven and dried at 80 °C for 8–10 h to obtain white particles of PECD. The PECD yield was approximately 60.1%.^10^

### 2.4 Synthesis of intermediate PEDSCD

**Fig. 3:**
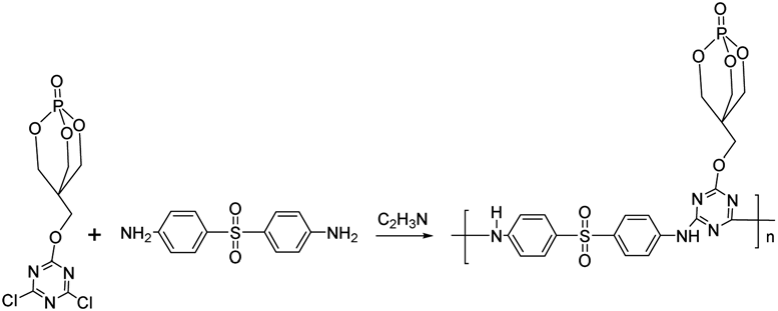
Synthetic route of PEDSCD.

The synthetic route of PEDSCD is shown in Fig. 3. Under nitrogen protection, 300 ml of acetonitrile was added in a three-necked flask with 0.1 mol PECD and 24.9 g (0.1 mol) DDS. The condensation reaction was carried out at 120 °C for 24 h, and then the heating was stopped. The obtained product was rinsed with acetone and acetonitrile three times. It was then placed in a vacuum oven, whose temperature was maintained at 80 °C. The product was dried in the vacuum oven for 10 h to obtain a white solid with a yield of 50.6%.

### 2.5 Purification of PEDSCD

Firstly, PEDSCD and 200 ml DMSO were placed in a round bottom flask at 80 °C, and it was stirred until the mixture was completely dissolved. After the solution became completely transparent, most of the DMSO was evaporated and the white solid was filtered out. Due to the high solubility of raw materials in acetonitrile, PEDSCD was insoluble in acetonitrile. Thus, it was separated based on the difference in solubility. The obtained white solid was analyzed by NMR and FTIR spectroscopy.

### 2.6 Preparation of PEDSCD/EP composites

The curing agent DDM and Teflon mold were placed in an oven at 80 °C for preheating for 5–10 min. According to the composition in Table 1, different amounts of PEDSCD were added into a 250 ml beaker containing 50 g EP. The mixture was stirred for 20 min in a constant temperature oil bath at 80 °C. Then, the curing agent DDM was added, and the mixture was poured into the mold to solidify and shape it.

**Table 1:** Composition of PEDSCD/EP composite samples (APP: ammonium polyphosphate)^a^

Sample	EP (wt%)	PEDSCD (wt%)	DDM (wt%)
EP-0	100	0	17.5
EP-1	95	5	17.5
EP-2	90	5 + 5 (APP)	17.5
EP-3	90	10	17.5
EP-4	85	15	17.5

a The amount of each component in the flame-retardant sample mentioned in the article is the mass ratio.

### 2.7 Characterization

#### 2.7.1 FTIR spectroscopy

The FTIR spectra of the generated pyrolysis gases of the samples was obtained by a Bruker Tensor 27 Fourier transform spectrophotometer equipped with thermogravimetric analyzer. These spectra were obtained in the range of 4000 to 550 cm^−1^ from the samples in KBr pellets. Furthermore, the FTIR analysis was performed at a resolution of 4 cm^−1^ in the range of 4000 to 550 cm^−1^. The thermogravimetric analyzer and FTIR spectrometer were connected by a quartz capillary at 300 °C.

#### 2.7.2 NMR spectroscopy

^1^H NMR (400 MHz) and ^31^P NMR (163 MHz) were recorded on a Bruker AVANCE NMR spectrometer. All the ^1^H NMR and ^31^P NMR data were obtained in d_6_-DMSO and referenced to the residual protonated solvent and phosphoric acid, respectively.

#### 2.7.3 Gel permeation chromatography (GPC)

GPC was performed on a Waters Breeze™ 2 HPLC system with a 2489 UV/visible detector and a 1515 isocratic HPLC pump. Both the column and the detector were maintained at 40 °C during the analysis.

#### 2.7.4 TGA

TGA was performed on a Mettler-Toledo TGA/DSC thermogravimetric analyzer under a nitrogen atmosphere. Approximately 5 mg of sample was weighted in an alumina crucible and heated from 50 to 700 °C at a rate of 20 °C min^−1^.

#### 2.7.5 Scanning electron microscopy (SEM)

SEM was performed on the cross-section of EP nanocomposites using a Hitachi SU8010 scanning electron microscope. The samples were coated with a layer of gold prior to observation.

#### 2.7.6 Cone calorimeter test

The flame retardant behavior was assessed on a Fire Testing Technology apparatus with a heat flux of 50 kW m^−2^ according to ISO 5660, and the size of the specimens was 100 × 100 × 1.2 mm^3^. All the samples were mounted on an aluminum foil and tested three times, and the average data was obtained.

#### 2.7.7 LOI measurement

The LOI value was measured on a JF-3 oxygen index meter (Nanjing Jiangning Analysis Instrument Co., China) in accordance with ASTM D2863. The specimens were prepared with dimensions of 130 × 6.5 × 3 mm^3^ by molding.

#### 2.7.8 Vertical burning test

The vertical burning test was conducted on a CZF-3 instrument (Nanjing Jiangning Analysis Instrument Co., China). The prepared specimens had a dimension of 125 × 12.7 × 3.2 mm^3^ in accordance with ASTM D3801.

#### 2.7.9 X-ray photoelectron spectroscopy (XPS)

The XPS data were obtained on a PHI Quantera II SXM at 25 W under a vacuum pressure lower than 10^−6^ Pa.

Elemental analysis methods and instruments: when determining the content of P elements in flame retardants, the digestion substance used is aqua regia, the measurement wavelength of P element is 213.618 cm^−1^. The test method refers to US EPA 3052:1996. The N element is tested by Kjeldahl nitrogen analyzer model KDN-04A.

## 3. Results and discussion

### 3.1 Structure and thermal stability of PEDSCD

**Fig. 4:**
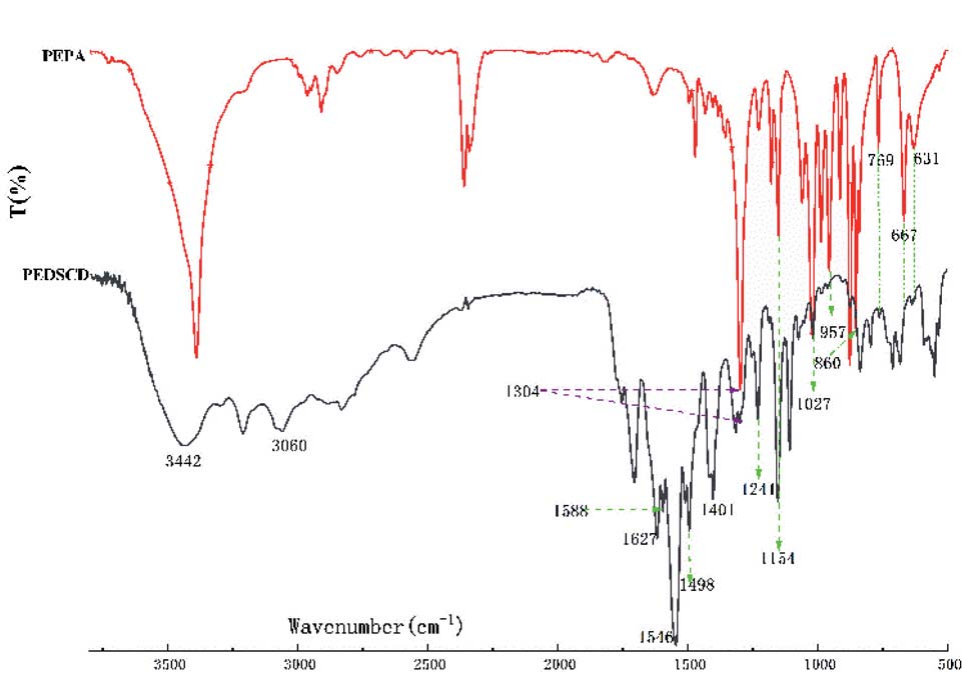
FTIR spectra of PEDSCD and PEPA.

Fig. 4 shows the FTIR spectra of prepared PEPA and PEDSCD samples. Here, the peak at 3442 cm^−1^ is the characteristic absorption peak of –NH_2_ from DDS. The absorption peaks at 1304 and 1027 cm^−1^ are attributed to the stretching vibration of P=O and P–O–C, respectively. Further, the peaks at 1154 and 959 cm^−1^ confirm the existence of –C(CH_2_)_4_ structure and –CH_2_–OH structure, respectively. The peaks at 860, 769, 667, and 631 cm^−1^ are the characteristic peaks of caged rings.^11,12^ The peaks at 2960 and 2905 cm^−1^ confirm the existence of –CH_2_ structure.^13,14^ These groups of peaks appear in the FTIR spectra of both PEPA and PEDSCD. In the spectrum of PEDSCD, the absorption peaks at 1546, 1627, 1241, and 1401 cm^−1^ confirm the existence of triazine ring structure on the molecular backbone,^15,16^ and the peaks at 1498 and 1588 cm^−1^ indicate that there is a benzene ring structure in the PEDSCD molecule. These absorption peaks confirm the synthesis of PEDSCD.

**Fig. 5:**
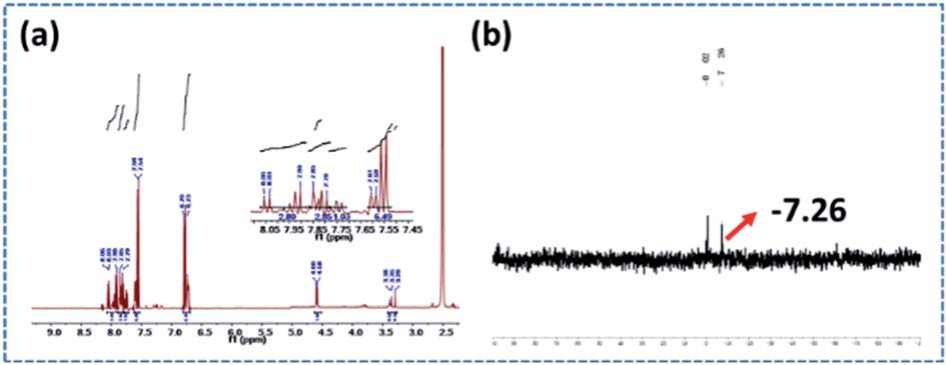
(a) ^1^H NMR spectrum of PEDSCD; (b) ^31^P NMR spectrum of PEDSCD.

The ^1^H and ^31^P NMR spectra of PEDSCD are shown in Fig. 5. In Fig. 5(a), the peaks at 4.58–4.60 ppm correspond to the hydrogen of CH_2_ on –O–CH_2_–C–.^17,18^ The peaks at 6.73–6.79 and 7.54–8.06 ppm correspond to the hydrogen on the benzene ring of DDM,^19,20^ while the peak at 3.29 ppm corresponds to the hydrogen in –CH_2_– at –C–CH_2_–O.^21^Fig. 5(b) shows the ^31^P NMR spectrum, the chemical shift of phosphorus in PEPA is −6.3–−7.1 ppm, and after the formation of PEDSCD, under the influence of the N element in the triazine structure, the chemical shift of phosphorus moves to a high field.^22^ Where the chemical shift of −7.26 ppm, corresponds to the absorption peak of phosphorus in the main chain of PEDSCD.

The elemental composition of flame retardant PEDSCD is shown in Table 2. During the synthesis process, the PEPA group is somewhat substituted, so when measured, the phosphorus content of the phosphorus element is low; and the presence of C=N and N–Ar leads to a higher nitrogen content in the flame retardant.

**Table 2:** Elemental composition of PEDSCD

Element	N (g kg^−1^)	S (g kg^−1^)	P (g kg^−1^)
Test value	204.5	75.2	5.022

**Fig. 6:**
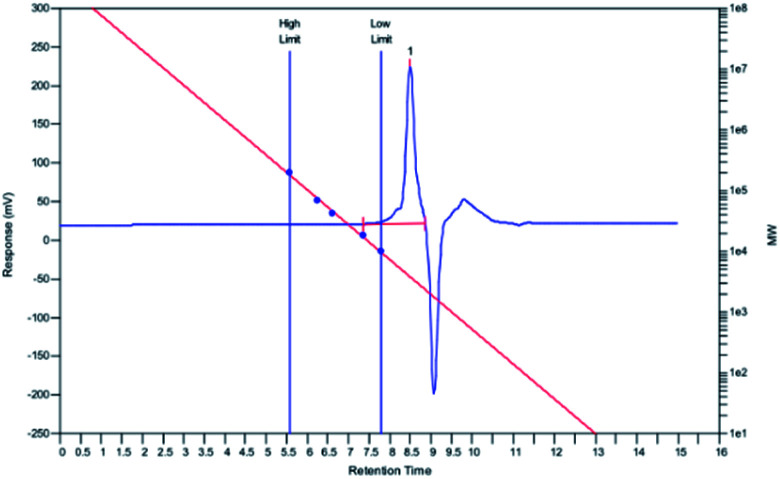
GPC of PEDSCD.

The molecular weight of PEDSCD was determined by GPC. Fig. 6 is the GPC test curve of PEDSCD, which are listed in Table 3. The number average molecular weight (*M*_n_), sticky molecular weight (*M*_w_), and polydispersity index (PDI) were 3810, 4008, and 1.05, respectively.

**Table 3:** GPC of PEDSCD

Element	*M*_n_	*M*_w_	PD
Test value	3810	4008	1.05

**Fig. 7:**
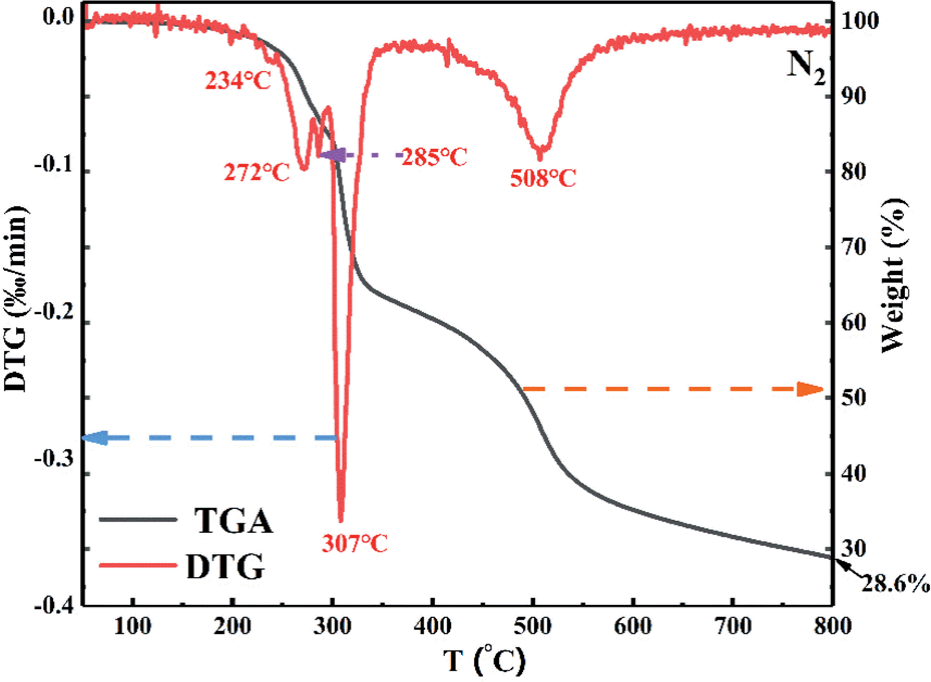
TGA and DTG curves of PEDSCD.

The thermal stability of PEDSCD was characterized by TGA, and the thermogravimetry/differential thermal analysis (TG/DTG) curve is shown in Fig. 7. It is evident that the PEDSCD begins to decompose at a low temperature. Before 580 °C, the PEDSCD has three temperature ranges where the rate of weight loss is relatively large. At 234 °C, the first peak appears in the DTG curve. At 272 °C, the second peak appears in the curve, where the remaining mass of PEDSCD is 90 wt%. When the temperature is increased to 285 °C, the third small peak appear, and the remaining mass of PEDSCD is 85 wt%. At 307 °C, the thermal weight loss rate of PEDSCD is the largest, where the DTG curve has a sharp peak, and the remaining mass of PEDSCD is 78.1 wt%. In the temperature range of 299–333.3 °C, the thermal weight loss is the fastest, and the mass loss is in the range of 83.6 to 64.7 wt%. At 800 °C, the mass of PEDSCD carbon residue is nearly 28.6 wt%. The sample begins to lose weight at approximately 200 °C, which is due to the low decomposition temperature of the triazine group on the main chain. At the same time, there is a sulfone group with high thermal stability on the molecular main chain, so the second weight loss rate of PEDSCD is very high. A large peak appears at 508 °C; the side group of the molecule is phosphate ester, which can reduce the decomposition rate of carbon residue. Between 546–800 °C, the mass loss of flame retardant is 10 wt%, and the thermal stability of carbon residue is better in the high temperature stage. The content of PEPA structure in PEDSCD molecule is extremely low, resulting in very low P content in the PEDSCD. During pyrolysis, the content of PEPA acting as an acid source is extremely low, which can esterify, cross-link, and aromatize the polyhydroxyl in the carbon source. The effect is weak, and the entire weight loss curve is relatively smooth. Further, the pyrolysis gas release process is uniform, which is beneficial to the formation of the carbon layer.

The thermal stability test results of PEDSCD in nitrogen atmosphere are shown in Table 4. It is observed that due to the introduction of triazine structure in the molecular backbone, the initial decomposition temperature of PEDSCD is much lower than that of PEPA. When the decomposition weight loss is 50%, the temperature of PEDSCD is 492 °C, and when the weight loss of PEPA is 50%, the temperature of the flame retardant is 467 °C. Overall, the results verify that the sulfone group on the main chain of PEDSCD helps to improve the thermal stability at high temperature.

**Table 4:** TGA parameters of PEDSCD under nitrogen atmosphere

Sample	*T*_*x*%_ (°C)				Residual char (500 °C, wt%)	Residual char (700 °C, wt%)
	(*x* = residual char, wt%)					
	99%	95%	90%	50%		
PEDSCD	180	254	274	492	48	32

### 3.2 Thermal stability of PEDSCD/EP composites

**Fig. 8:**
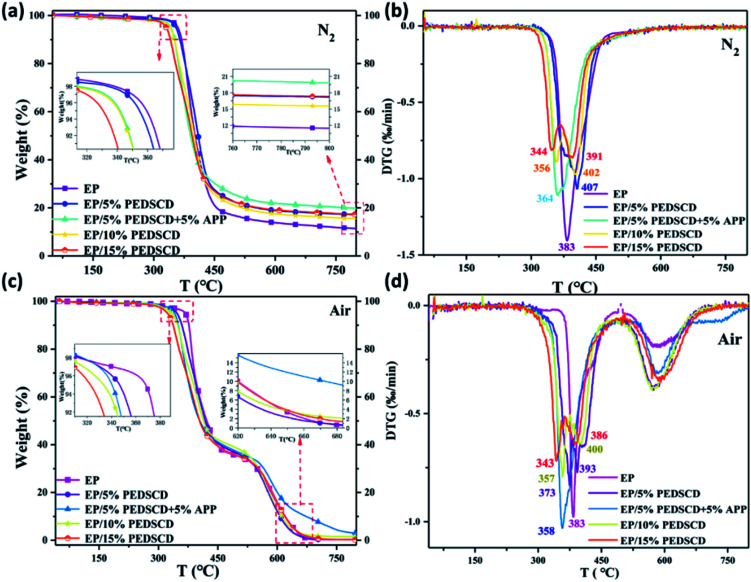
(a and b) TG and DTG curves of PEDSCD/EP under nitrogen atmosphere; (c and d) TG and DTG curves of PEDSCD/EP in air atmosphere.

The TG curves of five PEDSCD/EP cured products: EP-0, EP-1, EP-2, EP-3, EP-4, in nitrogen and air atmosphere are shown in Fig. 8. Table 5 lists the pyrolysis data of the samples in nitrogen and air atmosphere. In nitrogen atmosphere, all the samples have only one rapid weight loss stage. The initial pyrolysis temperature of the PEDSCD/EP sample is lower than that of the cured epoxy sample, which is inversely proportional to the amount of PEDSCD added. Before 440 °C, the weight loss rate of the sample EP-2 first decreases, while that of EP-0 decreases last. The TG curve of EP-0 is below the curves of other PEDSCD/EP samples. Further, it is confirmed that the addition of PEDSCD is effective before 440 °C. The pyrolysis process of EP matrix has a delaying effect. The weight loss curve of sample EP-2 is above the curves of other samples, verifying that APP can promote the flame retardant effect of PEDSCD/EP composite as APP and PEDSCD have a synergistic effect.

**Table 5:** Thermal degradation properties of PEDSCD/EP cured products^a^

Sample	*T*_onset_ (°C)		*T*_max_ (°C)		Residual char (500 °C, wt%)			Residual char (700 °C, wt%)		
					N_2_		Air	N_2_		Air
	N_2_	Air	N_2_	Air	Exp	Cal	Exp	Exp	Cal	Exp
EP-0	388	368	383	383	17		33	13		0.22
EP-1	355	346	407	373	23	19.0	36	18	13.9	0.4
EP-2	340	314	364	358	26	22.3	37	21	13.9	8
EP-3	333	317	356	357	21	21.1	38	16	14.6	2
EP-4	330	323	344	343	22	25.2	35	18	16.2	0.8

a *T*_onset_: 5% weight-loss temperature; *T*_max_: maximum weight-loss rate temperature; Exp: experimental value; Cal: calculated value.

There are two rapid weight loss stages in the pyrolysis process of PEDSCD/EP under air. The thermal oxidation stage occurs between 290–410 °C, while the carbon pyrolysis stage occurs between 560–640 °C. The values of initial pyrolysis temperature (*T*_onset_) and maximum pyrolysis rate temperature (*T*_max_) of PEDSCD/EP samples under air atmosphere are lower than those under nitrogen atmosphere. Table 5 lists the thermal degradation performance parameters of PEDSCD/EP cured products.

Fig. 8 and Table 5 show that the residual carbon content of PEDSCD/EP in air atmosphere at 500 °C is higher than that in nitrogen atmosphere. In air, the residual mass of EP-2 reaches 37 wt% at 500 °C and 8 wt% at 700 °C, while that of EP-0 at the same temperature (700 °C) is 0.22 wt%, which proves that the formed residual carbon layer of EP-2 is more thermally stable than that of EP-0 at 700 °C. In nitrogen atmosphere, the residual carbon content of EP-2 at 500 °C is 26 wt%, which is 4 wt% higher than the theoretical value. The residual mass of EP-2 is 9 wt% higher than that of EP-0. This indicates that the addition of PEDSCD promotes the increase of residual mass and improves the thermal stability of EP. In nitrogen atmosphere, the residual mass of EP-2 at 700 °C is 21 wt%, while the theoretical value is 13.9 wt%, and the residual mass of EP-0 is 13 wt%. It can be seen that the carbon residue structure of EP-2 at 500 °C has a strong protection ability for the matrix. In particular, the thermal stability of EP-2 in nitrogen atmosphere is significantly better than that of the non-flame retardant sample EP-0.

After the amount of PEDSCD exceeds 10%, the experimental value of the pyrolysis residue content of PEDSCD/EP is either equal or lower (and not higher) than the theoretical value. This is because when PEDSCD is added to the EP, it reduces the cross-linking density of PEDSCD/EP; the content of PEPA group in PEDSCD is very small, and the flame retardant effect of phosphorus is relatively low. PEDSCD mainly releases non-combustible gas from the triazine groups, which has a diluting effect and char-forming effect on the flame retardant. With the increase in the amount of PEDSCD, the interfacial compatibility between different components of the system becomes poorer, and the destructive effect of PEDSCD on the cross-linking density of the system becomes increasingly obvious. As the amount of PEDSCD increases, the experimental value of the pyrolysis residue content of the PEDSCD/EP becomes lower than the theoretical value. When PEDSCD is incorporated in PEDSCD/EP, the phosphorus content in the system primarily affects the quality of the pyrolysis residue in the system.

### 3.3 LOI and vertical burning test

**Fig. 9:**
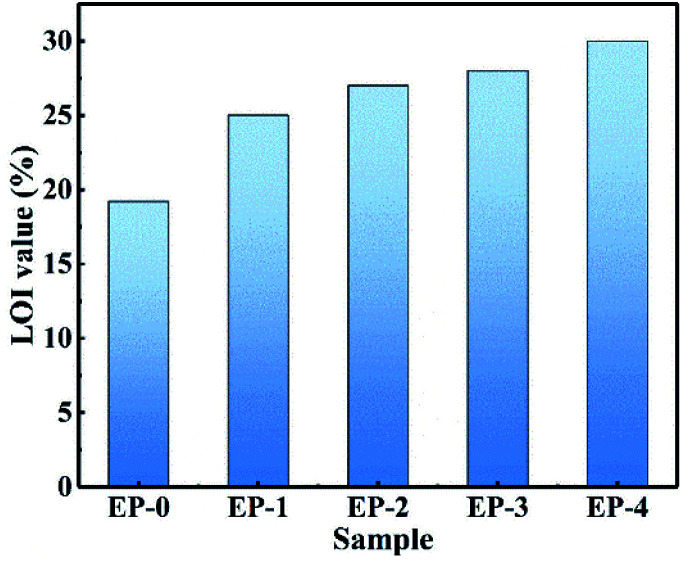
LOI histogram of PEDSCD/EP samples.

The LOI test and vertical combustion test were conducted to investigate the flame-retardant performance of the PEDSCD/EP samples. It is found that pure EP is easy to burn, and the LOI value is 19.2%. The combustion process is accompanied with the release of violent smoke and molten droplets. In several groups of flame-retardant epoxide samples, there is no dripping phenomenon. The LOI of EP-2 is 27, and its UL-94 rating reaches V-1, while the LOI of EP-3 is 28 and the UL-94 level reaches V-0. The results reveal that PEDSCD has good flame retardancy, and the flame-retardant performance becomes better when it is used in combination with APP (Fig. 9).

### 3.4 Cone calorimeter test

**Fig. 10:**
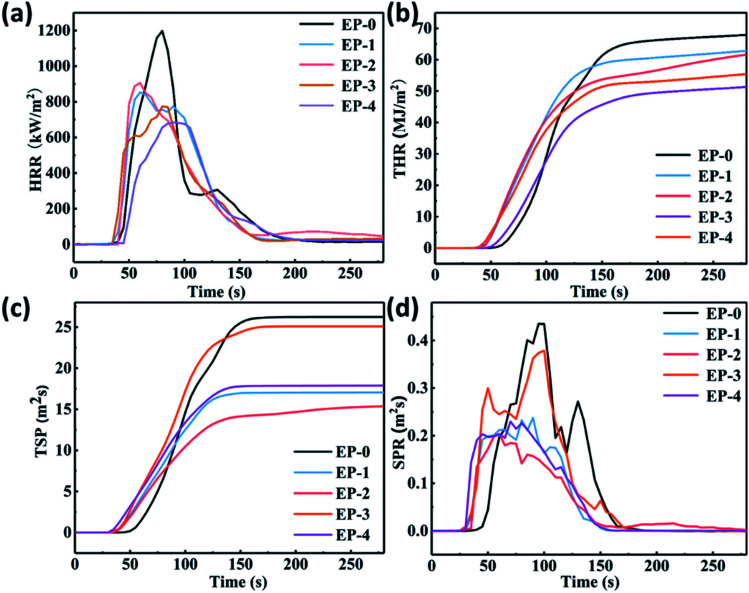
HRR, THR, SPR, and TSP curves of PEDSCD/EP.

Under the action of high temperature or flame, the acid source, carbon source, and gas source in the chemical IFR can quickly form a porous carbon barrier layer with heat insulation and barrier function through chemical reaction.^23–25^ The carbon layer can increase the resistance of heat exchange and material transfer between the base material and the combustion body, thereby reducing the pyrolysis rate of the material, changing the heat release rate (HRR) and the appearance time of the heat release peak, and reducing the quality of the combustible gas generated by the thermal decomposition of the polymer.^26–28^ The cone calorimeter test was used to evaluate and compare the flame -retardant performances of pure EP and PEDSCD. Fig. 10 and Table 6 show the results of the test, which include the time to ignition (TTI), total heat release (THR), HRR, peak HRR (PHRR), smoke production rate (SPR), total smoke production (TSP), average mass loss rate (AMLR) and time-to-flameout. It is clear that the PHRR and SPR of PEDSCD/EP are significantly reduced as compared to those of pure EP. The HRR curves of EP-0, EP-1, EP-2, EP-3, and EP-4 reach their peaks at 79.4, 60.1, 60, 83, and 82.7 s, respectively. The corresponding PHRR are 1188, 852.8, 905.7, 683.9, and 773.6 kW m^−2^ respectively. According to Fig. 10c, the SPR curve of the EP-0 cured product shows two peaks at 0.437 and 0.376 m^2^ s^−1^, while EP-1 (5% PEDSCD) shows two peaks at 0.209 and 0.236 m^2^ s^−1^, which are much lower than the peaks of EP-3 and EP-0. As the amount of PEDSCD increases, the peak heat release gradually decreases. When the addition amount of PEDSCD is 10% (EP-3), the PHRR is the lowest and the TTI is the longest. However, the SPR and TSP are higher than those of other flame retardants. Therefore, the flame retardant effect of the solidified phase carbon residue formed by EP-3 is not as good as that of other additives. EP-4 contains a certain amount of active hydrogen, which reduces the density of the cross-linked structure of the cured EP and affects the amount of smoke emitted.The PHRR of EP-1 is significantly lower than that of pure EP. The TSP of EP-1 is 17 m^2^, while that of pure EP is 18.5 m^2^, so EP-1 has a good smoke suppression effect. EP-2 has the same triazine structure content as EP-1, and the phosphorus content is increased by 1.59 wt%. The flashover time is significantly higher than that of EP-1, which can increase the escape time of people and improve the fire safety of materials.

**Table 6:** Combustion performance parameters of EP and PEDSCD/EP

Sample	TTI (s)	PHRR (kW m^−2^)	TSP (m^2^)	AMLR (g s^−1^)	Time-to-flameout (s)	UL-94
EP-0	21	1197.3	18.5	50.6	215	No level
EP-1	24	852.8	17.0	31.8	167	V2
EP-2	29	905.7	15.4	17.7	292	V1
EP-3	31	683.9	24.6	17.7	275	V0
EP-4	19	773.6	17.9	26.1	165	V1

When the chemical IFR system burns or emits heat, the phosphorus-containing compound and the nitrogen-containing triazine group compound work together to release incombustible gas or ammonia gas, promote polymer loss, and increase the residual polymer after combustion. The quality of the material promotes the formation of a dense foam expansion layer during the cross-linking and polycondensation of the viscous polymer material, which can effectively reduce the HRR and SPR while increasing the amount of non-combustible gas released by the system.^1–3^ The chemical components of the residual chars of EP to EP-4 cone calorimeter testing were investigated by XPS. The results are shown in Table 7. It is clear that the content of oxygen and nitrogen in the residual char is increased with the addition of PEDSCD. The increase is due to the presence of triazine groups in the flame-retardant cured material, which greatly increases the content of polyamide structures. The phosphorus content in the carbon residue increases with the addition of PEDSCD, indicates that the decomposition of PEDSCD produces phosphate, which is evenly distributed in the residual carbon, while reducing the pyrolysis rate of the material as well as the rate of smoke generation and escape. It can be seen that the composition of the carbon residue generated by the thermochemical reaction has a significant influence on the heat and smoke release when the material is burned. According to Table 7, the phosphorus content of the sample with 5% PEDSCD and 5% APP is lower than that of the sample with 5% PEDSCD. The XPS data of EP-0, EP-1 and EP-2 of P, O, C, and N elements are analyzed, and the results are shown in Table 8.

**Table 7:** Parameters of the carbon residue after the cone calorimetry test of EP and PEDSCD/EP

Sample	C 1s atom (%)	O 1s atom (%)	N 1s atom (%)	P 2p atom (%)
EP-0	88.25	7.95	3.63	0.17
EP-1	79.65	11.26	8	1.08
EP-2	79.75	13.65	4.32	2.28
EP-3	78.57	12.92	7.42	1.09
EP-4	83.15	9.12	5.13	0.9

**Table 8:** XPS results of the residual chars of EP, EP-1, and EP-2 samples

Sample	Element	Binding energy (eV)	Chemical bonding	Area
EP-0	C 1s	283.5	(C*H_2_–CH_2_)_*n*_	1700.7
		284.1	C graphite	9760.0
		285.8	CHO	3240.0
	O 1s	531.3	C=O	1649.9
		532.3	532.5 O 1s O–C·H_2_O	2271.9
	N 1s	397.4	Nitride	398.8
		398.5	NH_3_·BuNH_2_	63.4
		399.4	N–C R–NH_2_	508.9
		400.3	N–H or N–O	47.68
EP-1	C 1s	283.5	–(C*H_2_–CH_2_)_*n*_	1657.8
		284.1	C_6_H_5_X–CHO, CN, benzonitrile CN benzaldehyde CHO	7308.3
		285.8	C=O	5711.4
	O 1s	530.0	(C–O–C), YOOH (hydroxide)	1148.3
		531.6	OH–, P=O–, C=O	2501.2
		532.5	O–C·H_2_O	2529.9
	N 1s	397.4	Nitride	250.7
		398.3	–N= in a cyclic structure	455.5
		399.5	N–C, NH_2_–R	1224.3
		400.3	N–H or N–O	986.9
	P 2p	132.0	P=O	19.3
		133.9	N=P	267.5
EP-2	C 1s	283.5	–(C*H_2_–CH_2_)_*n*_	4202.1
		284.1	C_6_H_5_X–CHO, CN, benzonitrile CN benzaldehyde CHO	6592.8
		285.8	C=O	5402.7
	O 1s	530.0	(C–O–C) 530 YOOH (hydroxide)	1796.5
		531.6	OH–, P=O-, C=O	3692.5
		532.5	O–CH_2_O	2581.6
	N 1s	397.4	Nitride	150.3
		398.0	–N= in a cyclic	130.9
		399.5	N–C, NH_2_–R	725.4
		400.3	N–H or N–O	446.8
	P 2p	132.0	P=O	570.0
		133.9	N=P	145.6

**Fig. 11:**
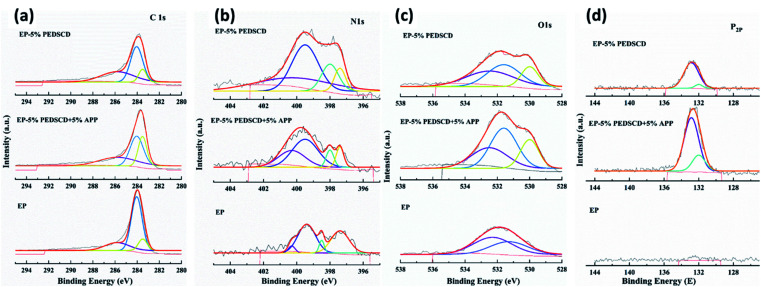
High-resolution XPS spectra of C, N, O and P.

It can be seen in Table 8 that compared with pure EP, except for the C element, the content of the other three elements in the flame-retardant EP samples increases to varying degrees. The relative content of oxygen is the highest, which is followed by that of nitrogen and phosphorus of PEDSCD/EP composites. Except EP-3, the other samples reveal the influence of PEDSCD on the thermochemical reaction of the PEDSCD/EP composites during the combustion process, and the XPS test data of EP-0, EP-1, and EP-2 are not changed significantly. The C 1s, N 1s, O 1s, and P 2p spectra of the samples are shown in Fig. 11. It is observed that the C 1s spectrum of both pure EP and PEDSCD/EP have peaks at 283.5, 284.1, and 285.8 eV. Among them, the area of the fitting peak of EP-2 at 283.5 eV accounts for the total area. The ratio is significantly higher than that of pure EP, and the area of the fitting peak at 284.1 eV is relatively small, which confirms that the fatty chain structure content in the carbon residue of the sample is relatively high, while the content of CO structure is relatively low, and the melt deformation ability during the combustion is large, which can effectively block the escape of smoke.^29,30^

The O 1s spectrum of pure EP has two peaks at 531.3 and 532.3 eV. After adding PEDSCD, the O 1s spectrum of carbon residue can be observed in three bands at 530.0, 531.6, and 532.5 eV, which can be attributed to the YOOH structure, P=O or C=O structure, and C–O–C structure,^31^ respectively, as shown in Table 7. The areas of peak at 531.6 eV and 531.6 eV in EP-2 are both higher than that in EP-1, so the relative content of phosphorus in EP-2 carbon residue is the highest. Further, the amount of hydroxyl oxygen compounds is higher, so its PHRR is slightly lower than that of EP-1, which means that PEDSCD and the carbon layer formed when APP is added can improve the heat insulation and suppress smoke. However, the carbon residue of pure EP does not have a peak at 531.6 eV. The N 1s spectrum of pure EP is dominated by the peak at 397.4 eV corresponding to the nitride structure, while in EP-2, the peak at 399.5 eV corresponding to the N–C and NH_2_–R structure dominates, which also confirms that the combustion of pure EP is extremely sufficient and the residual exists in the structure of the polyamide ring. Further, the relative content of N–C and NH_2_–R structure (at 399.5 eV) in EP-2 is higher. This result is consistent with the data in Table 6. The peak area of EP-1 and EP-2 at 400.3 eV is significantly higher than that of pure EP, which proves that the addition of PEDSCD changes the thermochemical reaction of the material, reduces the hydrogen content in the compound generated during combustion, and forms a carbon layer. This structure plays a protective role for the base material.

**Fig. 12:**
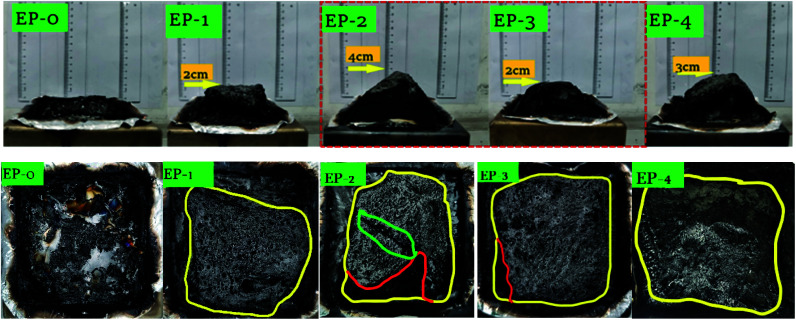
Digital images of carbon residue after cone calorimetry test of PEDSCD/EP samples.

The high-resolution spectra of C, N, O and P are shown in Fig. 11. The P 2p spectrum of PEDSCD/EP has two peaks. The peak at 132.4 eV indicates the presence of pyrophosphate and/or polyphosphate, while the peak at 133.9 eV can be assigned to the phosphorus atoms of the phosphazene ring,^32,33^ which confirms the observations already made for N 1s spectra. The relative relationship between the two peaks after fitting the P 2p spectrum is shown in Table 8, and it is found that the structure of pyrophosphate and/or polyphosphate in residual carbon of EP-2 is dominant, and the rest is phosphazene-like structure. The results of the TGA and the images of the residual carbon after the cone calorimetry test (Fig. 12) reveal that from the perspective of smoke suppression effect, EP-1 is better than EP-2.

Fig. 12 shows the digital images of the residues produced in the cone calorimeter test. It is clear that pure EP is almost completely combusted, and only a small amount of EP residue is formed after the cone test. In the top view of the carbon residue, the yellow line is the outline of the carbon residue, the red line is the boundary line between the foamed carbon residue layer and the fragmented carbon layer, and the green circled the crack outline of the EP-2 carbon residue layer. Compare these five samples the top view of the carbon residue layer after the cone volume test, you can see that the carbon residue layer formed by EP-2 is quite different from the square solidified sample, and there is a large crack on the surface, which confirms that the cone calorimetry test process the melt polycondensation process of EP-2 is not stable, and the entire melt structure is not uniform, which affects the heat release process and also leads to a decrease in the degree of compactness. However, the expanded carbon residue layer formed by the samples EP-3 and EP-4 that only added the flame retardant PEDSCD was relatively complete. Therefore, although the added APP increases the phosphorus content in the sample and promotes the increase in the amount of carbon residue, in the chemical expansion type resistance system, the compactness of the carbon residue is important for the heat release time and rate during the formation of the melt. And the total amount has a great influence.

Accordingly, with an increase in the amount of PEDSCD, the carbon layer becomes denser, which can effectively reduce the release of gas, it is in accordance with the cone calorimeter test results. Further, it is observed that when PEDSCD is used in combination with APP, the thickness of the carbon layer increases, indicating that PEDSCD can synergize with APP to improve the flame -retardant performance of EP.

## 4. Conclusion

In conclusion, a chemical IFR, called PEDSCD/EP, was synthesized by condensation polymerization of a new type of carbon source and phosphate ester. The thermal degradation and flame-retardant performance of IFR was qualitatively tested. The PEDSCD/EP samples with different amounts of flame retardants were prepared by curing with DDM. Compared with pure EP, the ignition time of samples with 5% PEDSCD/EP and 5% APP + 5% PEDSCD/EP were extended by 14% and 38% respectively. The flashover time of the sample with 5% PEDSCD and 5% APP was increased by 77 s, and the PHRR of sample containing 5 wt% PEDSCD was reduced by 30%. Based on the P 2p spectrum analysis of the carbon residue obtained after the cone calorimetry test of the flame-retardant sample, it was observed that after the thermochemical reaction of the sample with 5% PEDSCD, most of the phosphorous element products formed a phosphate structure, which effectively suppressed the formation of combustible gases and the escape of smoke. PEDSCD can also be used in combination with other flame retardants, or used in the flame retardancy of other polymer materials. All indicates PEDSCD has high research potential.

## Conflicts of interest

There are no conflicts to declare.

## Supplementary Material
